# Enhancing pediatricians’ engagement on social media: the role of board style questions

**DOI:** 10.15694/mep.2021.000177.1

**Published:** 2021-06-16

**Authors:** Kim Little-Wienert, Todd Chang, Rita Agarwal, Rachel Cramton, Karin Hillenbrand, Apurva Panchal, Wesley Stubblefield, John Mahan, Martha Wright, Lisa Donato, Latha Chandran

**Affiliations:** 1 Texas Children's Hospital; 2 Children’s Hospital Los Angeles; 3 Lucile Packard Children’s Hospital; 4 University of Arizona College of Medicine in Tucson; 5 East Carolina University Brody College of Medicine in Greenville; 6 University of Kansas Health System in Kansas City; 7 Infants' and Children's Clinic in Florence; 8 Nationwide Children’s Hospital; 9 Case Western Reserve University in Cleveland; 10 American Academy of Pediatrics in Chicago; 11 University of Miami Miller School of Medicine in Miami

**Keywords:** Social Media, Educational Measurement, Collaborative Education

## Abstract

This article was migrated. The article was marked as recommended.

**Background:** Social Media is used among medical professionals for collaborative education. Little is known about how case discussions prompt engagement.

**Objective:** To determine the association between item characteristics of board exam-style questions to social media engagement.

**Methods:** This was a prospective cohort study through the American Academy of Pediatrics (AAP) PediaLink FaceBook page, conducted in 2018 over 9 months. Items from the 2017 PREP® questions were ranked in difficulty, then rated in relevance to general pediatrics through content-expert consensus. Thirty-six questions were randomly posted on FaceBook and Twitter weekly. Independent variables included item difficulty rank, difficulty level (easy vs hard), relevance to general pediatrics, and word count. Outcome variables included percent correct responses and total comments under the post.

**Results:** More difficult questions were associated with fewer comments (rho=0.63, p<0.001) and lower correct response percentages (rho=0.39, p=0.02). Easy questions garnered more comments than hard questions (median 18 IQR 13-23 vs median 9.5 IQR 5-14, p=0.001). Correct response percentage was lower for hard questions (90% IQR 85-95% vs. 77% IQR 60-94%, p=0.04). Relevance to general pediatrics and word count did not affect engagement (p > 0.1).

**Conclusion:** Easier practice test items attracted more responses from pediatricians on social media, increasing engagement.

## Introduction

Social Media (SM) is used in medical education at the undergraduate and graduate medical education levels, using popular platforms including Twitter and FaceBook ( Admon *et al.*, 2019; Cheston, Flickinger and Chisolm, 2013; Sterling *et al.*, 2017; Nicolai *et al.*, 2017). Medical education efforts using these platforms have centered around their ability to create forums for online community discussion ( Nicolai *et al.*, 2017; Ranginwala and Towbin, 2018). Twitter has been used often at live conferences to spur synchronous or asynchronous discussion in an open, inclusive manner ( Desai *et al.*, 2018), while FaceBook is more visible in the medical literature as a medium for exclusive community discussions in closed groups ( Wright *et al.*, 2019).

The American Academy of Pediatrics (AAP) provides a variety of educational content, including board review-style questions within a program called Pediatric Review and Education Program (PREP). Since 1979, PREP has provided in print form, these questions and their detailed answers as self-directed learning materials. In 2000, PREP moved to an electronic version, and in 2017, SM campaigns to spur its use and awareness were introduced.

FaceBook has been used as an educational adjunct in healthcare simulation ( Ranginwala and Towbin, 2018), medical student training ( Sterling *et al.*, 2017; Nicolai *et al.*, 2017), and in a variety of disciplines including radiology ( Ranginwala and Towbin, 2018). Within the platform, an asynchronous discussion of relevant topics can be initiated either by faculty and attending-level physicians or by peers. The quality and depth of engagement within a discussion group dictates the richness of content in such social media platforms ( Rishika *et al.*, 2013). However, the factors that facilitate participant engagement in SM groups in a medical education setting are poorly understood.

The purpose of our study was to identify factors that enable enhanced engagement of participants in a social media learning platform. We hypothesized that greater the cognitive difficulty of the practice questions, the greater the engagement with the materials as evidenced by more participant comments and discussion. We also hypothesized that items with more relevance to general pediatricians would elicit more engagement as the majority of AAP members are general pediatricians.

## Methods

### Population and Setting

**Figure 1:**
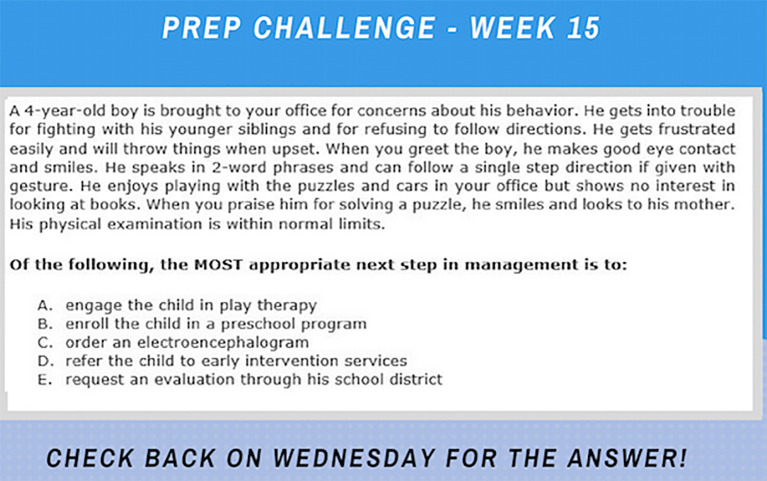
Screenshot of a typical case vignette

This was a prospective observational study conducted between February to October 2018 using the FaceBook and Twitter accounts for the AAP PediaLink group. PediaLink provides oversight over e-learning and educational technology offerings for the AAP; the SM team uses the PediaLink account to post information about AAP educational products including PREP. PREP, a practice board-style questions and answers set is updated each year for use by general pediatricians and pediatric subspecialists. All PREP questions are framed as a realistic case vignette followed by a multiple-choice answer format. As part of the SM promotion efforts, the AAP has been posting online through FaceBook and Twitter sample PREP questions since 2017. Subscribers to the PediaLink FaceBook Page or the PediaLink Twitter account are free to respond as they choose. **Figure 1** depicts a screenshot of a posted PREP question.

All pediatricians who had subscribed to the FaceBook group during the study period were eligible to participate in the study. There were no exclusion criteria. The study was deemed exempt by the AAP Institutional Review Board.

### Curation of PREP questions

We used the General Pediatrics PREP questions from 2017 for this study. There were 280 multiple-choice case vignette items developed and curated by the standard peer-review process through the AAP PREP Editorial Board. All test items had response data from more than 10,000 respondents for each item from previous subscribers or users of PREP. This enabled us to determine the percentage of correct respondents for all 280 items. Each PREP question was then ranked in difficulty from 1- 280, corresponding from the lowest correct response rate to the highest. Thus item 1 was the hardest and item 280 the easiest question.

We randomly selected 70 PREP test items from the overall 280 using stratified randomization. From each heptile rank in difficulty, 10 PREP questions were randomly selected using a random number generator ( http://www.random.org/); as an example, ten questions came from ranks 1-40, another ten from 41-80, etc. Then all 70 questions were randomized in order. The test item order is presented in **Table 1**.

Then, each item was assigned a scale on *relevance to a general pediatrician.* This was a de novo 7-point Likert scale for each item to quantify whether the case vignette portrayed a common, typical case seen in a general pediatrician’s office. Seven physicians - 4 general pediatricians and 3 pediatric specialists - rated all 70 item vignettes independently. The mean of all 7 rater scores became the *relevance* score for each test item.

### Protocol

An AAP PediaLink staff member (LD) posted a weekly PREP question in predetermined order beginning in February 2018. The PediaLink FaceBook page also had additional direct marketing information about AAP and PediaLink offerings, but no other case vignette discussions nor test items were posted during this study period. A pediatric specialist (RA) posted an open-ended, single sentence comment on the content of that question on a weekly basis; her identity as a study author was not known to the subscribers. Her comment was a ‘catalyst.’ The answer to the PREP test item was posted the following week alongside the new question for that week in the pre-determined order. The PediaLink Twitter page also mirror-posted the PREP questions but replies and re-tweets were not tracked for this study. No active recruitment strategies to either the FaceBook Page nor the Twitter account were added during this study period.

An interim analysis was planned at Week 36, and authors agreed *a priori* to stop the study if the analysis yielded significant results. Otherwise the protocol would continue until Week 70 with the final answer revealed on week 71.

### Variables of Interest

The primary independent variable was item difficulty, expressed both as an ordinal rank variable from 1 - 280. A rank of 1 was the most difficult, and 280 the easiest. Item difficulty was also dichotomized to difficult vs. easy, in which difficult items represented ranks 1 - 140 and easy 141 - 280. Secondary independent variables included the *relevance to a general pediatrician* score, expressed as a continuous variable between 1 and 7.

Our primary outcome variable was the total number of Face Book comments per item. We decided that this would be a quantitative marker of participant engagement with the posted material. Comments include responses of any length, including a simple letter response “c.” or a detailed explanation or question on either FaceBook or Twitter. If one participant posted 5 comments, all 5 comments were included. The catalyst comment from the study author was not included, and neither were comments posted after the answer was revealed. Secondary outcome variable included the correct response rate, calculated by dividing any correct responses divided by the total number of comments in both social media platforms.

### Data Analysis

We first documented a weekly temporal trend in the total number of comments on both FaceBook and Twitter for overall social media activity using Spearman Rank. Descriptive statistics were used to characterize comments and correct response rates for the items. Spearman Rank correlations were performed for continuous and ordinal independent variables of difficulty and relevance to a general pediatrician. A Mann-Whitney U test was done for the dichotomous independent variable: item difficulty. A mixed-method average measures intraclass correlation using a consistency type was performed for the 7 raters involved in the *relevance to a general pediatrician* score and 95 ^th^ confidence intervals reported ( Koo and Li, 2016).

## Results/Analysis

**Figure 2:**
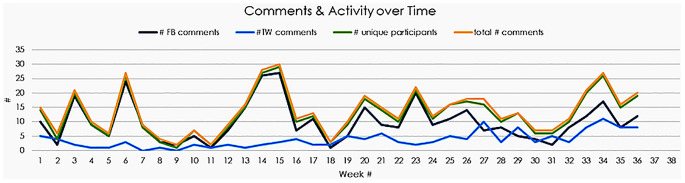
Overall social media activity

As described in the protocol, the study was discontinued following significant results recognized during interim analysis in week 36. **Figure 2** depicts overall social media activity during the weekly PREP test item protocol from February to October 2018; there was only a trend towards significance in increasing SM engagement (rho = 0.3, p = 0.07), which paralleled the gradual increase in social media followers: 4,999 to 5,322 Twitter followers and 3,862 to 3,914 FaceBook subscribers.

**Table 1** details the type of questions posted, the number of comments and the general pediatrics relevance score for all the test items. For the 36 test items posted on social media, the median number of comments was 12.5 (IQR 8.5 to 18.25) and the correct response rate had a median of 90% (IQR 74.1 to 100%). Among the 2017 PREP cohort whose correct response rate contributed to the difficulty rank assignments, the median correct response rate was 76% (IQR 67.25 to 83.5%). Our SM cohort had a significantly higher correct response rate than the 2017 PREP cohort (p = 0.005).

**Table 1:** Characteristics of the 36 questions used in this study

Q * #	Specialty Category	Difficulty Rank	Relevance to a General Pediatrician	FaceBook comments	Twitter comments	Unique Participants	# Correct Answers/# Incorrect Answers	Correct Response Rate (%)	2017 PREP Correct Response Rate (%)
**1**	Disorders of the Eye	80	6.57	10	5	14	9/5	64%	68%
**2**	Blood and Neoplastic Disorders	12	2.29	2	4	4	3/1	75%	48%
**3**	Ear, Nose, and Throat Disorders	248	6.71	19	2	20	20/0	100%	93%
**4**	Infectious Diseases	29	4.43	9	1	9	7/2	78%	54%
**5**	Sports Medicine and Physical Fitness	108	4.00	5	1	5	4/0	80%	74%
**6**	Behavioral and Mental Health Issues	175	6.57	24	3	26	24/2	92%	83%
**7**	Allergic and Immunologic Disorders	112	3.00	9	0	8	7/1	88%	74%
**8**	Infectious Diseases	79	4.86	3	1	3	3/0	100%	68%
**9**	Disorders of Cognition, Language, and Learning	107	5.43	2	0	1	1/0	100%	74%
**10**	Infectious Diseases	35	4.86	5	2	7	5/2	71%	56%
**11**	Pharmacology and Pain Management	4	3.29	1	1	2	1/1	50%	40%
**12**	Infectious Diseases	49	5.43	7	2	8	8/0	100%	63%
**13**	Adolescent Medicine and Gynecology	228	4.00	15	1	15	15/0	100%	89%
**14**	Cardiovascular Disorders	260	3.43	26	2	27	27/0	100%	95%
**15**	Disorders of Cognition, Language, and Learning	95	6.43	27	3	29	22/4	76%	71%
**16**	Endocrine Disorders	116	2.86	7	4	10	7/3	70%	75%
**17**	Sports Medicine and Physical Fitness	216	4.71	11	2	12	12/0	100%	87%
**18**	Blood and Neoplastic Disorders	13	3.14	1	2	3	3/0	100%	48%
**19**	Disorders of Cognition, Language, and Learning	138	5.43	5	5	9	5/2	56%	78%
**20**	Genetics and Dysmorphology	177	2.71	15	4	18	18/0	100%	83%
**21**	Musculoskeletal Disorders	94	5.57	9	6	14	8/6	57%	71%
**22**	Fetus and Newborn Infant	54	3.57	8	3	10	8/1	80%	64%
**23**	Skin Disorders	163	4.57	20	2	21	10/12	48%	81%
**24**	Poisoning and Environmental Exposure to Hazardous Substances	156	2.71	9	3	11	11/0	100%	80%
**25**	Growth and Development	56	7.00	11	5	16	8/8	50%	64%
**26**	Collagen Vascular and Other Multisystem Disorders	265	4.57	14	4	17	16/1	94%	96%
**27**	Ear, Nose, and Throat Disorders	194	4.29	7	10	16	14/2	88%	85%
**28**	Disorders of the Eye	145	4.00	8	3	10	9/1	90%	79%
**29**	Infectious Diseases	128	4.29	5	8	13	10/2	77%	77%
**30**	Research and Statistics	258	3.14	4	3	6	6/0	100%	95%
**31**	Renal and Urologic Disorders	62	2.29	2	5	6	6/0	100%	65%
**32**	Disorders of Cognition, Language, and Learning	142	5.86	8	3	10	9/1	90%	79%
**33**	Ear, Nose, and Throat Disorders	243	6.14	12	8	20	19/1	95%	92%
**34**	Critical Care	279	2.86	17	11	26	27/0	100%	98%
**35**	Respiratory Disorders	171	3.14	8	8	15	8/6	53%	82%
**36**	Sports Medicine and Physical Fitness	84	4.57	12	8	19	19/0	100%	69%

* Q=Question

More difficult test items were strongly associated with fewer comments (rho = 0.63, p < 0.001), and a correlation was found between difficulty rank and the correct response rate among 2018 social media respondents (rho = 0.39, p = 0.02).

When dichotomized, *hard* test items attracted a median of 9.5 (IQR 5 to 14) comments, while *easy* test items attracted significantly higher median of 18 (IQR 13 to 23) comments (p = 0.001) per item. Even among the social media respondents, hard questions yielded a 77.4% (IQR 60 to 94%) correct response rate, which was significantly lower than the easy questions’ 97.5% rate (IQR 92.5 to 100%, p = 0.04).

Among the 7 raters for the 70 selected items, the *relevance to a general pediatrician score* demonstrated a strong ICC of 0.910 [95%CI 0.874 to 0.939]. However even with such reliable ratings of relevance to a general pediatrician, we found no significant associations between items that were more *relevant to a general pediatrician* and to either total number of comments (rho = 0.3, p = 0.1) or to the correct response rate (rho = -0.2, p = 0.2). Incidentally, word count of the items was inversely associated with relevance to a general pediatrician (rho = -0.35, p = 0.003), indicating subspecialty questions were wordier.

## Discussion

Our data show that easier case vignette questions posted among pediatricians yielded more responses and engagement than more difficult questions. SM has been used in educational endeavors as a method to transcend temporal and geographical constraints and to facilitate an ad hoc community ( Admon *et al.*, 2019; Cheston, Flickinger and Chisolm, 2013; Sterling *et al.*, 2017; Nicolai *et al.*, 2017; Melvin and Chan, 2014).

While the characteristics of the comments was not analyzed for this study, the majority of engagement to the multiple-choice case vignettes used in this study consisted of posting the letter choice of a response. On occasion, we saw dialogue-based conversations, but these tended to be rare, particularly for more difficult questions.

Our hypothesis was incorrect. We had anticipated that more difficult questions would engender greater conversations and dialog among the participants; but they did not. The behavior that was demonstrated on our SM platform is consistent with the literature on SM and social comparison theory ( Vogel *et al.*, 2014). While the content in our study was not particularly charged or controversial, the significant drop in comments with more difficult questions suggest that our adult learner subjects may have been reluctant to post an incorrect response to potentially avoid social embarrassment or judgment. Studies on anonymity in classroom settings indicate that anonymity may lower the barriers for reluctant students to speak or to actively participate ( Barr, 2017). Given that FaceBook subscribers were not anonymous, the lower comment rate seems indicative of reluctance to err in such a public forum.

Another interesting observation was that the correct response rate among our SM cohort was substantially better than the rate for the 2017 PREP test takers. There are several possible reasons for this finding. It may suggest self-censorship among participants who did not post a comment to avoid social embarrassment as discussed earlier, thereby driving the correct response rates higher. Another possible reason for the higher performance by the SM group might be their open access to the web where they could potentially search for the right answers before posting on the platform. As savvy users of online learning, this group is more likely to be engaging in such “just in time learning” compared to the traditional test takers.

We did not find an association with *relevance to a general pediatrician* to the level of engagement and responses on SM. While we do not know the specialization of the AAP PediaLink subscribers, based on AAP data, general pediatricians consist of approximately 75% of AAP members. The literature on SM suggests that a complex relationship exists between SM engagement and the degree of controversy of a topic ( Garimella *et al.*, 2018). Relevance and ‘topicality’ is known to affect responses and comments online outside of medical education ( Chen, Shang and Li, 2014); however, we did not see substantial changes in comments based on relevance to general pediatrics. It is also possible that general pediatrics questions would have been more highly commented if we had collected a larger sample size. To find any significant association at a predicted rho of 0.28 would have required a sample size of 97, which would have taken almost 2 years to collect in this study design.

There were several limitations to this study. First, the study team did not have control over any other social media posting, including marketing or event notification posts. While no particular marketing ‘surges’ were noted, it is possible that extraneous materials influenced participation outcomes. The number of subscribers during the time period of the study was projected to increase, but the actual subscriber number was not available to us. Finally, although the data were aggregated from two social media platforms, we did not examine whether certain characteristics influenced commentary depending on the platform.

## Conclusion

Enhancing social media engagement by participants is a complex multifactorial process. As the difficulty of posted test items increases, the correct response rate among pediatricians on social media and the number of posted comments and engagement decreases significantly. SM education groups - in pediatrics or in any specialty or discipline - can purposefully curate test item postings to garner different levels of engagement and discussion among participants.

## Take Home Messages

• Easier board exam-style questions posted on social media were associated with more responses or comments posted from pediatricians, increasing engagement significantly from engagement with harder questions.
• Harder board exam-style questions posted on social media were associated with lower correct response percentages from pediatricians.
• Question relevance to general pediatrics and question word count did not affect the number of comments or level of engagement seen from pediatricians on social media.
• Medical professionals aiming to improve engagement and discussion among participants on social media for collaborative education using test questions should consider the level of difficulty of the questions.

## Notes On Contributors

**Kim Little-Wienert, MD, MEd** is Assistant Professor of Pediatrics and Pediatric Emergency Medicine Fellowship Associate Director at Texas Children’s Hospital and Baylor College of Medicine in Houston, TX.

**Todd P. Chang, MD MAcM** is Associate Professor of Pediatrics at the University of Southern California and Children’s Hospital Los Angeles (CHLA) in Los Angeles, CA. He serves as the Director of Research and Scholarship within the Division of Emergency Medicine and Associate Medical Director for the Las Madrinas CHLA Simulation Center.

**Rita Agarwal, MD** is Clinical Professor of Anesthesiology at the Stanford School of Medicine in Palo Alto, CA and the President of the Society of Pediatric Pain Medicine.

**Rachel E. M. Cramton, MD** is Associate Professor of Pediatrics and Pediatric Residency Associate Program Director at University of Arizona and Banner University Medical Center in Tucson, AZ.

**Karin Hillenbrand, MD MPH** is Professor of Pediatrics at Brody School of Medicine, East Carolina University in Greenville, NC.

**Apurva Panchal, MD** is Associate Professor of Pediatrics at University of Kansas Health Center in Kansas City, KS in the Division of Pediatric Critical Care Medicine.

**Wesley Stubblefield, MD, MPH** is a pediatrician at Infants’ and Children’s Clinic in Florence, AL.

**John D. Mahan, MD** is Professor of Pediatrics and Chair of Faculty Development for Nationwide Children’s Hospital and Ohio State University College of Medicine in Columbus, OH. He is also the Chair for the American Academy of Pediatrics Pedialink Editorial Board.

**Martha S. Wright, MD, MEd** is Professor Emerita in the Department of Pediatrics at Case Western Reserve University in Cleveland, OH.

**Lisa Donato** is the Education Activities Coordinator at the American Academy of Pediatrics in Chicago, IL.

**Latha Chandran, MD, MPH** is Executive Dean and Founding Chair in the Department of Medical Education at University of Miami Miller School of Medicine in Miami, FL.
