# Supplementary material for: Enhancing pediatricians’ engagement on social media: the role of board style questions
Source: MedEdPublish (2016). 2021 Jun 16;10:177. [Version 1] doi: 10.15694/mep.2021.000177.1 (PMC10939574; doi:10.15694/mep.2021.000177.1)
Supplement: Peer review history

# Reviewer report: Reviewer response for version 1

**DOI:** 10.21956/mep.20441.r31663

**Published:** 2021-06-23

**Authors:** Helena Filipe

**Affiliations:** 1 Hospital of the Armed Forces/PL-EMGFA

This review has been migrated. The reviewer awarded 4 stars out of 5 Thank you for the opportunity to review this paper, which I read with great interest.There has been an increasing attention towards the use of social media (SM) in medical education, recently propelled for the pandemic. These challenging times have underscored the need to develop collaborative, supportive, informal, safe social learning spaces. Fostering wellbeing and interpersonal support within a community of learning has been prioritised to learn effectively.Kudos for a step forward on advancing consistent ground for the use of SM in medical education. The use of widely known daily life social applications sprung have far from the academia accurate requirements. The authors make the case, that more than a compartmental field, the scientific method can have multiple applications and be the selected methodology for any field of inquiry. Thank you for describing comprehensively to the point of replication, your research. The results are quite interesting and open the wide range of potential applications of SM in knowledge building in contemporary innovative learning environments. Educators, planners, and providers will be interested to read this piece, which highlights the importance of their own CPD to effectively embrace and apply new facilitation skills to meet learners needs (1). They are expected to gear the rich opportunities offered by technology towards building multifaceted learning experiences. We tend to highlight and wonder about three results of this investigation:-While engendering less responses, and still considering the anonymity issue, could more difficult questions suggest coupling a small-groups (guided) reflective learning space, as a follow-up?- As an effectiveness determining factor for CPD activities, practice-based learning has been associated with adult learning principles. Results invite to better understand the absence of a proportional engagement level with topics’ relevance. This could start by expanding the sample, as Authors propose. -While yielding a higher level of engagement, might be interesting to explore the potential use of easier questions as drivers to build supportive and safe digital learning environments and how this could relate with learning improvement.The 4 messages left by the Authors summarise well the study purpose, methods and what was found. 1. Filipe HP, Mack HG. Teaching through social media. Ann Eye Sci 2019;4:28. Doi: 10.21037/aes.2019.07.02

Reviewer Expertise:

NA

No decision status is available

# Reviewer report: Reviewer response for version 1

**DOI:** 10.21956/mep.20441.r31664

**Published:** 2021-06-22

**Authors:** Anita Samuel

This review has been migrated. The reviewer awarded 3 stars out of 5 This article explores creating engagement in social media for educational purposes. Considering the increase in the use of platforms like Twitter for medical education, it is helpful to understand what enhances participant engagement. This information can enable others to create more impactful social media posts. Below are some suggestions to strengthen this article:- Your first hypothesis mentions ‘more participant comments’ and ‘discussion’. Your conclusion reiterates this as ‘levels of engagement’ and ‘discussion’. In the study, you are using the number of comments to indicate the level of engagement. But discussion, or depth of engagement, is not explored in this study. You might want to be careful in your use of the term discussion since it confuses the focus of this study.- If only pediatricians subscribed to the Facebook group were eligible to participate in the study, how did you include Twitter comments and participation on Twitter? Was Twitter also covered by the IRB?- You state in your Protocol that for Twitter, replies and retweets were not tracked. But you were counting ‘comments’ posted on Twitter. How are you differentiating between comments and replies on Twitter? - You have spelled dialogue in different ways in the text. It might be good to use one consistent spelling. - What is the impact of this study? Will the findings of this study influence AAP posts in the future?

Reviewer Expertise:

NA

No decision status is available
